# Syndromic surveillance of population-level COVID-19 burden with cough monitoring in a hospital emergency waiting room

**DOI:** 10.3389/fpubh.2024.1279392

**Published:** 2024-03-28

**Authors:** Forsad Al Hossain, M. Tanjid Hasan Tonmoy, Sri Nuvvula, Brittany P. Chapman, Rajesh K. Gupta, Andrew A. Lover, Rhoel R. Dinglasan, Stephanie Carreiro, Tauhidur Rahman

**Affiliations:** ^1^Manning College of Information and Computer Sciences, University of Massachusetts-Amherst, Amherst, MA, United States; ^2^Halıcıoǧlu Data Science Institute, University of California, San Diego, San Diego, CA, United States; ^3^Department of Emergency Medicine, UMass Chan Medical School, Worcester, MA, United States; ^4^School of Public Health & Health Sciences, University of Massachusetts Amherst, Amherst, MA, United States; ^5^Infectious Diseases and Immunology, University of Florida, Gainesville, FL, United States

**Keywords:** syndromic surveillance, ambient sensing, cough counting, emergency medicine, respiratory illness

## Abstract

Syndromic surveillance is an effective tool for enabling the timely detection of infectious disease outbreaks and facilitating the implementation of effective mitigation strategies by public health authorities. While various information sources are currently utilized to collect syndromic signal data for analysis, the aggregated measurement of cough, an important symptom for many illnesses, is not widely employed as a syndromic signal. With recent advancements in ubiquitous sensing technologies, it becomes feasible to continuously measure population-level cough incidence in a contactless, unobtrusive, and automated manner. In this work, we demonstrate the utility of monitoring aggregated cough count as a syndromic indicator to estimate COVID-19 cases. In our study, we deployed a sensor-based platform (*Syndromic Logger*) in the emergency room of a large hospital. The platform captured syndromic signals from audio, thermal imaging, and radar, while the ground truth data were collected from the hospital's electronic health record. Our analysis revealed a significant correlation between the aggregated cough count and positive COVID-19 cases in the hospital (Pearson correlation of 0.40, *p-*value < 0.001). Notably, this correlation was higher than that observed with the number of individuals presenting with fever (ρ = 0.22, *p* = 0.04), a widely used syndromic signal and screening tool for such diseases. Furthermore, we demonstrate how the data obtained from our *Syndromic Logger* platform could be leveraged to estimate various COVID-19-related statistics using multiple modeling approaches. Aggregated cough counts and other data, such as people density collected from our platform, can be utilized to predict COVID-19 patient visits related metrics in a hospital waiting room, and SHAP and Gini feature importance-based metrics showed cough count as the important feature for these prediction models. Furthermore, we have shown that predictions based on cough counting outperform models based on fever detection (e.g., temperatures over 39°C), which require more intrusive engagement with the population. Our findings highlight that incorporating cough-counting based signals into syndromic surveillance systems can significantly enhance overall resilience against future public health challenges, such as emerging disease outbreaks or pandemics.

## 1 Introduction

The act of coughing is part of the body's defense system that uses automatic reflex mechanisms to clear airways of foreign substances, irritants and excessive mucus. Cough and related complaints have been recognized as a cardinal symptom or syndromic signal of respiratory illnesses, such as the SARS-CoV-2, influenza and influenza-like illness, asthma, chronic obstructive pulmonary disease (COPD), and lung cancer. Recent progress with cough assessment has led to improved diagnosis and more effective disease management for individual patients (1, 2). However, cough-related signals have been largely absent in population-level disease monitoring and public health surveillance. This is primarily due to lack of efficacious, scalable and contactless sensing infrastructure to gather cough event information (e.g., total occurrences at a location) from specific target populations.

In this work, we present an ambient sensing platform to capture different syndromic signals (e.g., daily cough counts) from the emergency department (ED) waiting room of a large hospital within a large metropolitan area. The deployed sensor platform *Syndromic Logger* is the next-generation contactless sensing platform (3). The *Syndromic Logger* platform captures non-speech audio with a microphone array for the detection of cough and speech events. With a built-in thermal camera, it can continuously capture thermal video to detect number of people in the hospital waiting areas (as a measure of waiting room crowd size). Lastly, with a ultra-wideband radar sensor, the platform can also capture human movements. In this work, we specifically focus on the significance of aggregated cough count as a syndromic signal for COVID-like illnesses. We collected sensor data over a period of four months and obtained ground truth data from electronic health record systems (EHR). Our results demonstrate that aggregated cough count is a strong indicator of total COVID burden within the hospital. Compared to aggregated fever count, a widely used syndromic indicator for population-level epidemiological models of respiratory diseases, we found stronger associations between total cough count and total COVID-19 case counts. Moreover, we show that the automatically captured syndromic data from *Syndromic Logger* in a waiting room can be used to develop a regression model for daily counts of COVID-19 cases in a hospital emergency clinic. Inclusion of such automatic syndromic signals/data using historical data and manually extracted information from EHRs in a regression model boosts the performance of daily COVID-19 case prediction. Overall, the paper highlights the capability of automatic, passive and contactless syndromic sensing for population-level COVID-19 burden monitoring in a hospital setting.

### 1.1 Primer on syndromic surveillance and the need for contactless syndromic sensing

As the world manages the staggering global public health crisis of COVID-19 and begins to move toward a “new normal,” our vulnerabilities to another outbreak of SARS-CoV-2 (or an equally devastating pathogen) are ever apparent. The ability to keep society safe and functional in the event of a resurgence is of paramount importance (4). The state-of-the-art disease monitoring and public health surveillance by the US Center for Disease Control and Prevention (CDC) primarily relies on aggregated reports from sentinel reporting sites including hospitals and selected outpatient clinics. However, there exists a substantial time lag in the reporting of such data (e.g., 7–14 days reporting lag time for influenza-like illness) (5, 6). The lack of real-time information on the infection dynamics and symptom dynamics of the target population is a fundamental gap that limits our ability to monitor and forecast disease trends to mobilize early interventions. Smart and connected syndromic surveillance with state of the art sensor systems to capture objective syndromic signals (e.g., cough, fever) unobtrusively from target population to enhance predictive intelligence and pandemic resilience strategies represent a prime opportunity. Such a syndromic computing framework can help to mobilize a rapid public health response, limiting the spread of infection, and consequent morbidity and mortality.

## 2 Related work

### 2.1 Syndromic surveillance for COVID pandemic

While syndromic surveillance has been long utilized in public health, it is an emerging area in computational epidemiology, due to the accessibility of high-resolution data sources. This paradigm aims to gather general symptom-related information (prior to any clinical diagnosis) of an infectious disease to allow for rapid responses. Several recent works aimed to achieve such goals especially in the context of the recent COVID pandemic using both active and passive monitoring of syndromic signals. Examples of active monitoring include self- reported body temperature and presence of symptoms relevant to COVID using a mobile application (7, 8); symptom and demographic data from people searching about COVID symptoms through a chatbot (9); symptom and testing data using internet and phone surveys (10); and voluntarily-reported symptom data (11). These approaches require active participation from the target population, may be clinically unreliable due to self-reporting, and may not capture subsets of vulnerable populations (e.g., older and less technology savvy demographics). Examples of passive monitoring include monitoring of emergency room activity in hospitals (12, 13), monitoring occupancy, and crowd size (14). These approaches might be subject to reporting delays, require active human effort (e.g., manually measuring and logging data) and may fail to capture objective and relevant syndromic signals.

### 2.2 Cough based syndromic surveillance for COVID

Current cough-based COVID-19 detection methods predominantly focus on individual diagnoses. These approaches utilize traditional machine learning (ML) techniques, including Logistic Regression, Support Vector Machine (SVM), Random Forest, and Gradient Boosted Trees, to classify COVID-19 from audio data (15–19). Additionally, deep learning models have been employed, encompassing RNN/LSTM-based classifiers (20) and CNN-based classifiers (21–26). However, these methods are primarily designed for individual-level COVID-19 detection and do not extend to population-level monitoring. Moreover, studies have revealed limitations in the practical application of these techniques for accurate, real-world, individual COVID-19 diagnosis (27).

To address these limitations, it is desirable to capture crowd level syndromic signals in a passive, contactless and automated manner. In addition, for genuine public health impact, syndromic surveillance platforms need to be scalable and to provide objective and real-time metrics informative of the total burden of specific disease burden in the target population or community.

## 3 Materials and methods

### 3.1 Study overview

This study was reviewed by the Institutional Review Board (IRB) at the University of Massachusetts Amherst. The study collected information about anonymous syndromic event in the room and aggregate daily data about the hospital testing metrics. As no individual level data or individually identifiable information was collected, UMass IRB determined that the research project is not human subject research and does not meet the definition of human subject research under federal regulations [45 CFR 46.102(d)]. The UMass IRB determination document is attached with this submission (ref #237; May 4, 2021).

**Figure 1:**
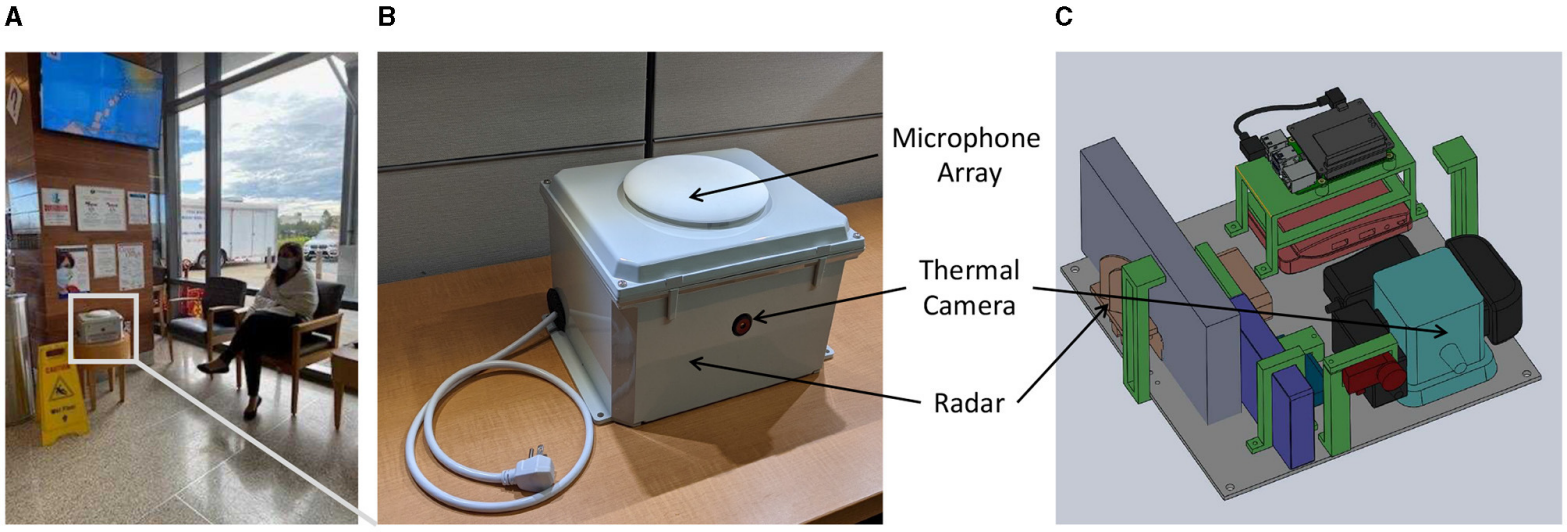
**(A)** Deployment location sample with an actor simulating the patient's sitting location; **(B)** top view of the ***Syndromic Logger*** device from outside **(C)** 3D-render of internal components of the ***Syndromic Logger*** device.

We deployed our device in the emergency department (ED) waiting room of a large, tertiary-care, academic medical center with a census of over 135,000 emergency visits per year. Figure 1 shows the layout of our waiting room, which includes the direction of typical foot traffic and seating locations and our sensor location and orientation. A written placard containing information on the objectives and protocol of the study was placed right next to the device. The ED operates 24 h a day, seven days a week. The waiting room space is occupied by hospital staff (nurse, patient care technicians, and security personnel), adult and pediatric patients, and accompanying visitors during the triage process. On busier days, patients and visitors may spend several hours in the waiting room prior to a bed becoming available in the main treatment area, which is in a separate location from the waiting area. We placed our sensor in the ED waiting room from August 1, 2021 to November 30, 2021, and then again from March 1, 2022 to April 30, 2022 for continuous syndromic data collection. Within the emergency department waiting area, the specific location for our device placement was determined based on the availability of power outlet, maximal capture area of thermal camera with its limited field of view, and high signal to noise ratio audio data recording. Routine checks of the device were performed during the data collection process to ensure proper positioning in the waiting room.

### 3.2 Syndromic surveillance platform

**Figure 2:**
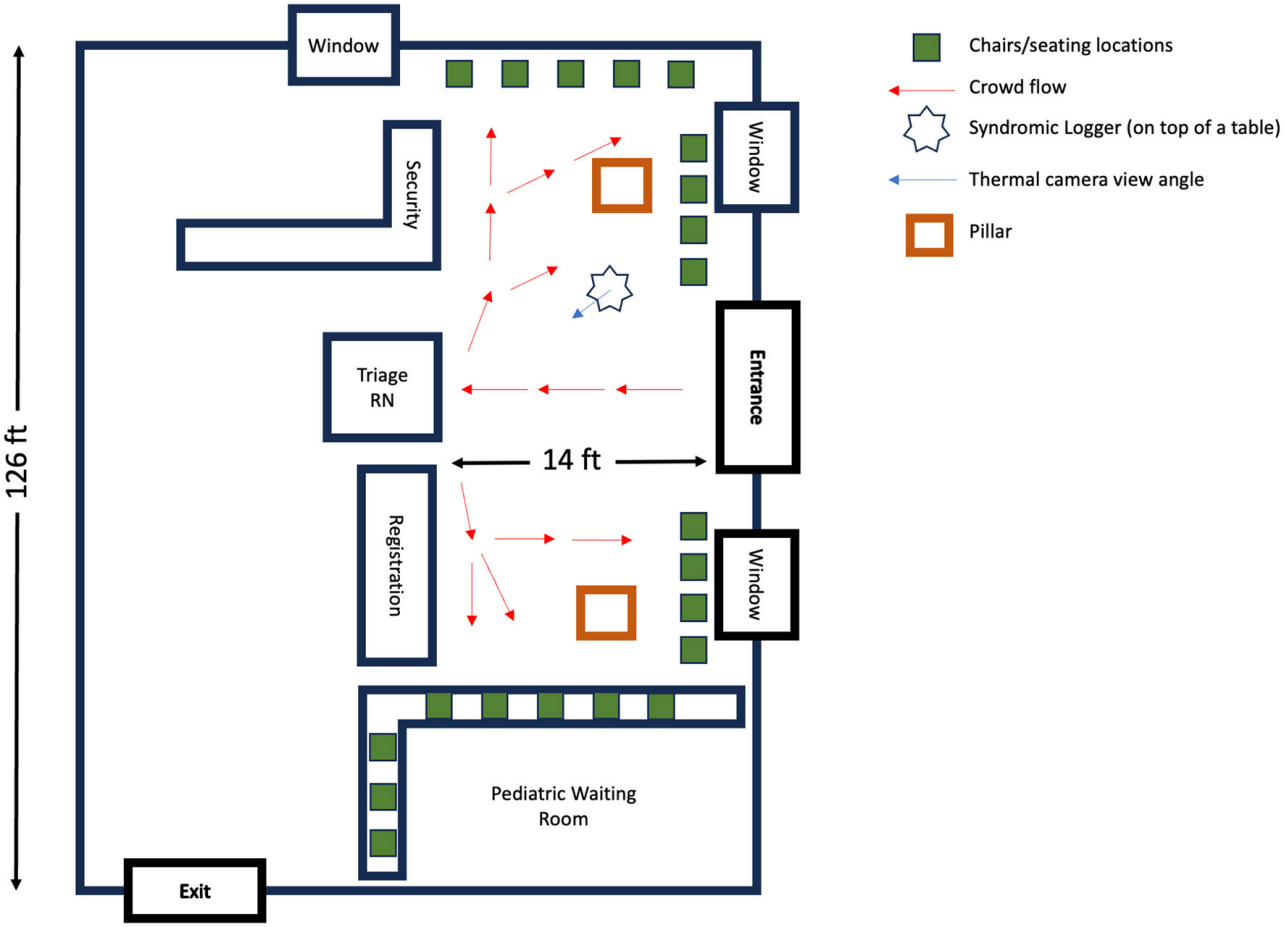
Emergency department waiting area: a schematic overview. This diagram illustrates the spatial layout and foot traffic flow in the Emergency Department's waiting room. Red arrows depict the usual movement patterns of patients: entering through the main entrance, proceeding to the triage nurse's desk and/or registration area, followed by moving to the designted seating locations.

Our *Syndromic Logger* platform (depicted in Figure 2) integrates a range of sensors and hardware components (microphone array, thermal camera, radar sensor, and Raspberry PI). It also incorporates a software system that leverages multiple machine learning models and ensures secure data storage. We provide detailed descriptions of the various hardware and software components and discuss the security and privacy aspects of the platform below.

#### 3.2.1 Hardware

Our ***Syndromic Logger*** platform includes the following hardware modules:

- ReSpeaker Microphone Array V2.0: A microphone array containing four microphones (28).
- SEEK CompactPRO: An inexpensive thermal camera (29) that can be used for people counting using thermal video.
- Intel Neural Computing Stick: A hardware platform (30) that is used for accelerating Deep Neural Network computation.
- PulseON 440 UWB radar: A radar sensor for monitoring movements and motions in the deployment area (31).
- Raspberry PI: A fully Linux-based embedded platform used for managing the sensors and processing and storing the data securely in real-time.

#### 3.2.2 Software system

##### 3.2.2.1 Audio processing

We developed our speech and cough recognition classifier using the dataset and model architectures described in Al Hossain et al. (3). For the construction of the cough and sneeze recognition model, we utilized the dataset sourced from FluSense-data (32), which was also used by the authors of Al Hossain et al. (3). To ensure consistency, we adhered to the data augmentation procedure outlined in Al Hossain et al. (3) and generated corresponding training, testing, and validation datasets.

For cough detection, we used the VGGish model (33). The VGGish was trained on AudioSet data (34). AudioSet is a massive collection of Youtube videos containing 10 seconds of weakly-labeled video segments; it includes 70 million videos collected from YouTube. The VGGish architecture is a variant of VGGNet architecture (35), with only the last layer changed and the Local Response Normalization (LRN) layers changed with BatchNormalization layers (33). To use the model with audio signals, we used the same dataset and augmentation pipeline from FluSense authors (3) and converted each 1 second long audio snippet into log-mel spectrograms of size 96 × 64.

Table 1 shows the performance of our cough model in different environments compared to the model used in prior work (3). We considered three environments: testing data without any augmentations, testing data with speech inserted in it, and testing data with hospital noise collected from YouTube. We also used the same dataset as Al Hossain et al. (3), which is the real-world hospital data containing cough and non-cough sounds. The list of non-cough sounds includes sounds that are abrupt in nature, similar to cough, such as door-slamming and dropped objects. The original model in Al Hossain et al. (3) had many false positives for these sounds. As evident in Table 1, our model works robustly in all testing conditions and outperforms the original model in Al Hossain et al. (3), achieving the best results during testing with real-world data samples even in the case when the training data did not include any such real world data.

**Table 1:** Performance comparison of our cough classifier(VGGish) with previously proposed cough classifier (3) in different acoustic settings.

**Testing sound type**	**Current model (VGGish)**			**Al Hossain et al**. **(****3****)**		
	***R (%)***	***P (%)***	***F1 (%)***	***R (%)***	***P (%)***	***F1 (%)***
No background noise	91.5	91.5	91.5	90.2	90.2	90.2
With speech	87	85.5	86	82.4	82.3	82.4
With hospital noise	87	88	86	84.5	85.4	84.4
With all augmentations	89.5	90.5	89.5	87	87.3	86.9
FluSense dataset (3)	93.1	93.2	93	89	87	88

For deployment, we first converted the models to 16-bit floating point precision model and then deployed the optimized model using OpenVino (36) framework. We used Intel NCS (version 2) to process the audio data stream in real time.

##### 3.2.2.2 Thermal data processing

We captured thermal video data from SEEK Thermal Pro camera. The resolution of our camera was 320 × 240 pixels, and our capture frequency was 5 frames per second.

Similar to the authors of Al Hossain et al. (3), we used a trained Faster-RCNN model (37) to detect the number of people from the video data. With the trained model, extract bounding boxes containing people and then use the bounding box areas to extract various features from the video data.

##### 3.2.2.3 UWB radar data processing

We used PulseON 440 UWB radar in the monostatic mode for collecting UWB radar data. The radar operates within bandwidths of 3.8 to 4.8 GHz with a center frequency of 4.3 GHz. It has a transmitter antenna that emits millions of very short duration pulses. These pulses are reflected both by stationary objects and moving objects. A co-located receiver antenna receives these backscattered signals and multiple scans of such received signals are stacked together to form an image representation, which is also known as a “radargram.” One dimension of the image corresponds to different range bins, which are generated by collecting reflected signals with varying time of flight. This time of flight, multiplied by the speed of light, estimates the round trip distance to a specific bin. The second dimension represents time, often referred to as slow time. Objects or humans in motion exhibit distinct signatures that fluctuate or traverse different range bins over the course of time.

To extract movement-related features, we removed the static clutter part of the radargram caused by static objects from the radargram. To achieve this, we first calculate the mean for each range bin of the radargram and then subtract the mean from each radargram. Afterwards, the radargram contains only the signals caused by movements from non-static elements in the environment (mostly people). After clutter removal, we apply the Fast Fourier Transform to each range bin to get a Fourier spectrum from each range bin.

#### 3.2.3 System security and preservation of privacy

Finally, to store all of the saved data in a secure manner, we used a 2-phase encryption scheme to store the data in a hard drive attached to our system. In the first phase, a random key is generated, and this key is used for encrypting captured data (i.e., audio snippet, video clip, or radar data) using symmetric encryption schemes. Using symmetric encryption ensures that our CPU load is low during this data chunk encryption process. After that, this randomly generated key itself gets encrypted with a public key, and it gets stored with the encrypted captured data. To decrypt data snippets, at first, we use a private key that is only available to us to decrypt the symmetric encryption key that was stored with the data snippet, and then we use that symmetric key to decrypt the stored sensor data. This 2-phase encryption scheme ensures that our data is secure, and in the case of theft or vandalism, data in our platform remains inaccessible without the private key.

As the US Federal Health Insurance Portability and Accountability Act (HIPAA) of 1996 forbids collecting any privacy-sensitive data within the scope of a non-HIPAA compliant framework, we explicitly did not save any speech data during our deployment. To omit these speech data from our saved dataset, we built the same model as the authors of Al Hossain et al. (3), which was shown to be very effective in a real-world deployment. During runtime, we detected whether we had speech content in each 1-sec slice of audio data. We only saved data for future analysis if it didn't contain any speech. Otherwise, we skipped saving the data to preserve user privacy. Prior to deployment, various aspects of our research protocol, including security, privacy and data storage policies, were thoroughly scrutinized by an Institutional Review Board (IRB) committee. Following careful deliberation, the IRB deemed the data collected in this study to not qualify as human subjects research.

### 3.3 Ground truth data collection

For ground truth data, aggregate daily data points were abstracted from electronic health record (EHR) and managed using REDCap (Research Electronic Data Capture) tools (38) hosted at the University of Massachusetts Chan Medical School. The ground truth data includes daily information about the ED patient volume, wait time, number of viral tests (COVID and non-COVID) ordered, and the number of positive viral tests (COVID and non-COVID).

Of note, COVID testing is done for a variety of reasons in the hospital (e.g., for diagnosis in symptomatic patients, to screen asymptomatic patients before a procedure, or before entry into a group care setting). For the purpose of this study, we excluded COVID tests ordered on asymptomatic patients for screening purposes, as their volume was more likely related to hospital policy and other factors as opposed to changes in community prevalence of SARS-CoV-2 infections. Symptomatic testing includes both immunoassays (commonly referred to as “Rapid” tests) and polymerase chain reaction-based (PCR) assays. We collected the following ground truth data fields.

- **Number of people**: This number of patient visits the emergency department (ED) for a given day.
- **Average wait time**: The average time an ED patient waited prior to being placed in a bed in the main treatment area
- **Any COVID test**: The total number of COVID -19 tests ordered on symptomatic ED patients. This number includes COVID tests as ordered within a Respiratory Viral Panel (RVP) and standalone COVID tests.
- **Positive COVID**: The total number of positive symptomatic ED COVID-19 tests. This includes tests ordered as part of Respiratory Viral Panel (RVP) and non-RVP).
- **Positive flu**: The total number of positive ED Flu Type-B using RVP testing methods.
- **Positive RSV results**: The number positive ED RSV (Respiratory Syncytial Virus) tests using RVP tests.
- **Fever (> 38°C)**: The total number of unique ED patients that had a fever recorded on their triage vital signs: fever is defined as temperatures greater than or equal to 38° degrees Celsius.

### 3.4 Feature extraction

In our study, we utilized our *Syndromic Logger* platform to gather syndromic signal data from audio, thermal, and UWB radar. We extracted the following features from the collected data.

#### 3.4.1 Audio-based features

We process ambient audio using an on-device machine learning model and used the following features for analysis:

- **Total cough count** (Total cough): The total daily cough count detected by our sensor platform.
- **Total speech count** (Total speech): This feature is the total number of speech snippets detected by our system on a daily basis.

#### 3.4.2 UWB radar-based features

The Ultra-Wideband (UWB) radar data captures movement-related features from the radar signals as described earlier. We extracted the following features for each day.

- **Filter bank features**: Human movement generates relatively low frequency responses and broadband radar signals, while machinery such as a fan and air conditioning units generate high frequency monotonic signals. Consequently, the spectral analysis of the radar signal can allow us to look at different types of movements captured by the UWB radar. We take a filter bank approach to quantify the energy in different frequency ranges or filter banks. To be specific, the cut-off frequencies associated with the filter banks are respectively 0.25–0.50 Hz, 0.5–0.75 Hz, 0.75–1.0 Hz, 1.0–1.25 Hz, 1.25–1.5 Hz, 1.50–1.75 Hz, 1.75–2.0 Hz, 2.0–2.25 Hz, 2.25–2.5 Hz, 2.5–2.75 Hz, 2.75–3 Hz. We observed in our analysis that human movement is associated with low frequency filter banks.
- **Total energy**(*tot*_*energy*): The total energy in the radar signals capture the total human movement related activities that occur in the waiting room. A crowded waiting room where the occupants are moving will consequently increase the magnitude of this total energy feature.
- **Standard deviation of energy per bins** (*std*_*energy*): We also extract the standard deviation of total average energy in different range bins which captures a measure of waiting room crowd dispersion.

#### 3.4.3 Thermal camera-based features

We captured thermal video data from SEEK Thermal Pro camera. The resolution of our camera was 320 × 240 pixels, and our capture frequency was 5 frames per second. Similar to prior work outlined here (3), we used a trained Faster-RCNN model (37) to detect the number of people from the video data. With the trained model, extract bounding boxes containing people and then use the bounding box areas to extract various features from the video data.

- **Person time** (Person-Time): Upon detecting the number of people in the waiting room based on the total number of bounding boxes in every frame (captured at a rate of 5 frames per second), we aggregate the total number of bounding boxes for the entire day to derive the “Person Time” feature. This Person-Time feature informs the model about crowd density.
- We did not extract body temperature feature from the thermal camera. This is primarily because of the relatively high temperature measurement error range (i.e., low precision) of the specific affordable thermal sensor that we used. More sophisticated (and costlier) thermal cameras exist in today's market that can reliably estimate human body temperature in a contactless manner. In fact, these thermal cameras are used in airports and transit stations to screen travellers with specific symptoms. We did not incorporate such an expensive equipment in our device considering the high cost and to maximize scalability of our syndromic surveillance system.

## 4 Results

### 4.1 Correlation analysis: syndromic signals and population-level disease metric

Table 2 presents the Pearson correlation coefficients between different sensor-based syndromic signals captured by our device and ground truth population-level disease burden metric extracted from the hospital's EHR system. which includes the number of people counted per day and total positive COVID, Flu, and RSV patient count.

**Table 2:** (a) Pearson Correlation between different syndromic features and Positive SARS-CoV-2, Influenza and RSV counts; *p*-value in parenthesis.

**Modality**	**Features**	**Positive COVID**	**Positive Flu**	**Positive RSV**
Audio	Total Cough	**0.40(< 0.001)**	–0.11(0.30)	**0.27(0.01)**
	Total Speech	0.20(0.06)	0.03(0.80)	0.00(1.00)
Thermal	Person-Time	–0.06(0.56)	0.00(0.98)	–0.07(0.53)
	num_peaks	–0.20(0.06)	0.25(0.02)	0.05(0.63)
	std_energy	0.18(0.10)	0.23(0.03)	0.19(0.07)
Radar	tot_energy	0.15(0.15)	–0.23(0.03)	0.17(0.12)
	0.0–0.25 Hz	0.13(0.23)	–0.16(0.14)	0.13(0.21)
	0.25–0.50 Hz	0.13(0.22)	–0.23(0.03)	0.15(0.15)
	2.50–2.75 Hz	0.15(0.16)	–0.23(0.03)	0.17(0.13)
	**# of People**	**Fever(**>38 **C)**	**Positive COVID**	**Positive Flu**
# of People	-			
Fever(>38 C)	**0.45(< 0.001)**	-		
Positive COVID	**0.28(0.01)**	0.22 (0.04)	-	
Positive Flu	0.04(0.72)	0.15 (0.16)	–0.02(0.86)	-
Positive RSV	0.07(0.51)	0.07(0.51)	**0.38(< 0.001)**	–0.17(0.12)

(b) Pearson correlation coefficient between different infections and waiting room crowd-related variables extracted from the hospital EHR systems. The *p*-value is reported within the parenthesis. The bold values are *p*-values which are ≤ 0.01 (which is considered as the threshold for significant *p*-value).

**Figure 3:**
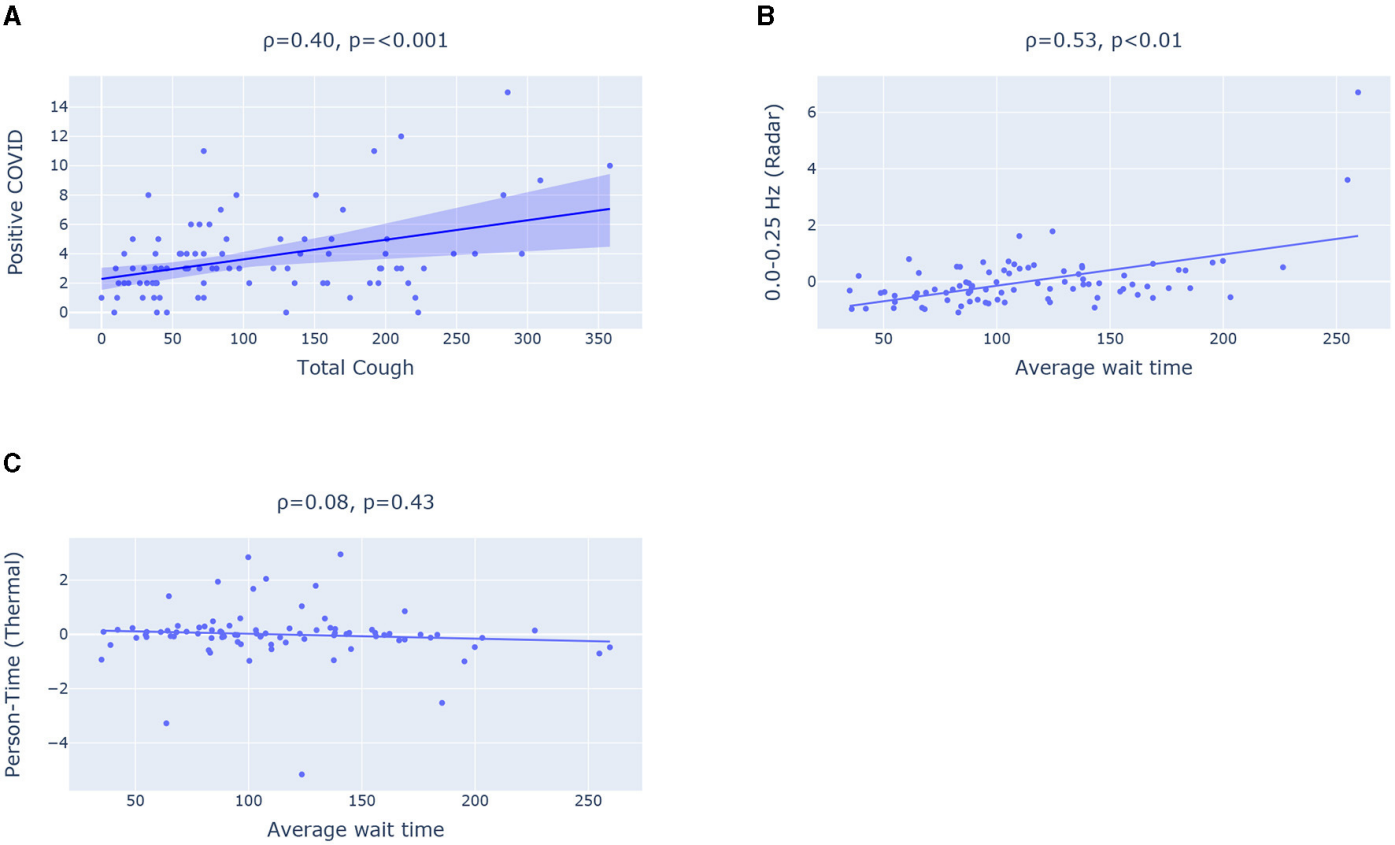
**(A)** Scatter plot showing correlation between Total cough and positive COVID. **(B, C)** Scatter plot highlighting Pearson Correlation between Average wait time (from EHR) and two features extracted respectively from **(B)** radar and **(C)** thermal camera.

**Key Observation 1: Total daily cough count has statistically significant correlation with positive COVID-19 case count**. When examining the correlation coefficients, we observe the most significant correlation between total daily COVID positive patients and the total number of coughs captured by our device (.40 with a *p*-value of 0.0001). This relationship is visually depicted in Figure 3A. Moreover, we also observe relatively high correlations between total cough and total daily positive RSV patients (0.27 with a *p*-value of 0.01). However, we did not observe any strong correlation with positive influenza count. This can be attributed to the fact that flu has generally more variable with the presence or absence of respiratory symptoms (e.g., some people have only gastrointestinal symptoms or muscle aches without cough).

To quantify time series trends with adjustment for serial correlation, multivariable models with negative binomial distribution and robust (Huber-White “sandwich” errors) were used. Additional sensitivity analyses were performed using bootstrapped errors with 2,000 replicates. All exploratory factors with *p* < 0.20 were taken into multivariable models, with model building guided by lowest AIC/BIC. “Day of week” was forced into all models regardless of significance. All tests were t-tailed, with α = 0.05. Analyses used Stata 17 (College Station, TX). To quantify the association between sensor-based metrics and positive SARS-CoV-2 tests, multiple cough-based measures were assessed, including “total coughs;” “total coughs” normalized to ln(low-band radar signal, 0.25–0.50 Hz); and “total coughs” normalized to thermal-sensor person-time.

The results from multivariate time series models are shown in Table 4. For each 13% increase (95% CI: 6% to 20%) in captured cough sounds (standardized to the lowest frequency radar bound output) there was a one-unit increase in positive SARS-CoV-2 tests, with adjustments for day-of-week.

**Key Observation 2: Total number of people visiting ED waiting room and total daily RSV count has a statistically significant correlation with positive SARS-CoV-2 tests**. Table 2 shows the correlation between the ground-truth variables. We can see high correlation coefficients between daily positive COVID patient count and the number of patients with positive RSV (0.38 with a *p*-value of 0.0003). This could be because the viruses peaked in the community around the same time and because people were co-infected (39, 40). Additionally, we can see that the number of people, and the number of positive COVID patients has a correlation coefficient of 0.22. Another important metric, fever > 38^*o*^*C*, which is often used an screening measure for detecting COVID, showed a stronger correlation with the number of people in the waiting room compared to the number of positive COVID cases. Specifically, we found correlation coefficient of 0.22 between fever and total COVID case count. In contrast, the correlation with number of people is 0.45, which is much higher than the value for positive COVID tests.

**Key Observation 3: The syndromic features extracted from radar (e.g., 0.0–0.25 Hz filter band energy feature) achieves statistically significant correlation with reference daily average wait time (a measure of crowd density) extracted from EHR. However, the Person-Time feature extracted from the thermal camera fails to show any statistically significant relationship with the average wait time in the waiting room as illustrated in** **Figures 3B**, **C**. The radar has a significantly greater sensing coverage area and can capture any movement in circle with 5 meter radius. The thermal camera, on the other hand, can capture thermal images with a 320 × 240 pixel resolution and a 32^*o*^ field of view. The limited field of view impact the thermal camera's ability to capture the entire waiting room crowd and consequently the estimated Person-Time from the limited field of view thermal images fail to capture the waiting room crowd behavior such as average wait time.

We can also observe that 0.0–0.25 Hz filter band energy feature and std_energy achieved a statistically significant correlation (of correlation coefficient of 0.58) as well. All these point to the fact that radar-based features can effectively inform COVID burden model about average wait time and crowd density (as a high average wait time indicates a crowded emergency waiting room).

### 4.2 Modeling COVID burden using sensor-based features

We aim to demonstrate the effectiveness of incorporating cough count as a feature in modeling COVID burden in the hospital, emphasizing its value as a syndromic signal. First, we develop baseline models that do not incorporate any sensor-based features. Subsequently, we show the utility of sensor-based features, particularly cough count, in enhancing the modeling of COVID-related statistics compared to the baseline. We evaluate the models using Mean Average Error (MAE) and correlation coefficient. Table 3 summarizes the different feature sets we use for the models. We chose the weekday index, a categorical variable that represents the day of the week, as our first baseline feature set B1. The remaining baseline feature sets (B2–B4) include positive COVID case counts from up to three previous days, enabling models to perform autoregression with historical data. We explored different models with baseline feature set and random forest yields the best performance when trained on baseline feature sets. We can see the results in Table 3. Among all the baseline feature-based model, the random forest trained on B4 feature set achieves the best performance with a correlation coefficient ρ of 0.13 and an *MAE* of 2.14. However, compared to the sensor-informed syndromic feature-based models, the baseline models achieves a significantly lower performance.

**Table 3:** (a) List of features in baseline and sensor-based feature sets, (b) Leave one day out cross-validation results for different models with different feature sets for three target variables: total COVID-positive cases, COVID cases as a percentage of hospital visits, and COVID cases as a percentage of number of tests done.

**Feature set**	**List of features**						
B1	Day of week						
B2	Day of week, positive COVID (previous 1 day)						
B3	Day of week, positive COVID (previous 2 days)						
B4	Day of week, positive COVID (previous 3 days)						
S1	Day of week, 0.0–0.25 Hz, Total Cough						
S2	Total cough, Total speech, std_energy						
**Model**	**Feature Set**	**Total COVID**		**COVID / People ratio**		**COVID / Test ratio (%)**	
		**MAE**	**Pearson corr**	**MAE**	**Pearson corr**	**MAE**	**Pearson corr**
Random forest	B1	2.1	–0.24	0.95	-0.32	4.63	–0.25
	B2	2.08	0.25	0.89	0.25	4.85	–0.02
	B3	2.2	0.19	0.96	0.17	5.09	–0.1
	B4	2.14	0.13	0.92	0.16	4.55	0.02
	S1	1.8	0.41	0.76	0.47	4.30	0.26
	S2	1.78	0.43	0.75	0.49	3.65	0.38
Linear regression	S2	1.91	0.29	0.82	0.34	3.99	0.24
Poisson Regression	S2	1.89	0.29	0.8	0.34	3.99	0.22
Grad-Boost	S2	1.77	0.45	0.75	0.53	3.66	0.43
SVR	S2	1.66	0.42	0.76	0.37	3.86	0.36

To assess the performance of sensor-based features compared to the baseline features, we selected three highly correlated input features for modeling the count of positive COVID cases: “Total Cough,” “Total Speech,” and “std_energy” (correlation coefficients of 0.40, 0.20, and 0.15, respectively). We conducted experiments employing various models, including Linear models (Linear Regression, Poisson Regression), Tree based models (Random-Forest, Gradient boosted trees), and Support Vector Machines with an “RBF” kernel. The Gradient-Boosted trees achieve the best results in terms of Pearson Correlation ρ (0.45), while the Support Vector Regression (SVR) model achieves the best *MAE* of 1.66. Overall, models using syndromic features (i.e., S1 and S2) from the *Syndromic Logger* platform substantially outperform the models using baseline features for the task of predicting the total COVID positive patient count.

In addition to the total number of COVID patients per day, we also modeled the prevalence of COVID as a percentage. To normalize the COVID count, we considered two scenarios: (a) the percentage of COVID-positive cases among the number of ED waiting room visitors, and (b) the percentage of COVID-positive cases among the total number of tests conducted. In both cases, our models utilizing sensor-based syndromic features exhibited significant improvements over the baseline models. For predicting the ratio of Total Positive COVID cases to the total number of people, our best model achieved a correlation coefficient of 0.53. It is significantly better compared to the baseline, which reached a correlation coefficient of 0.25 (with B2 feature set). Similarly, for predicting the percentage of COVID-positive patients among the total number of tests performed, our best model achieved a correlation coefficient of 0.43, in contrast to the baseline results of a correlation coefficient of 0.02 (with B4).

Furthermore, our analysis with Multivariable Negative-Binomial model (as demonstrated in Table 4) reveals a statistically significant relationship between the number of COVID positive tests and total cough normalized by radar based crowd density metric (i.e., a proxy variable for total cough per person time). In fact, the total cough metric was found to be a strong indicator and was the only syndromic variable that yielded a statistical significance in this multivariate negative-binomial analysis. We also observed that for each unit increase in cough-per-person there's a 86 increase in positive SARS-CoV-2 tests.

**Table 4:** Multivariable negative binomial model quantifying relationship between SARS-CoV-2 positive tests, and cough-based syndromic signals (IRR, incidence rate ratio (exponentiated coefficients).

**Exploratory factor**	**IRR**	**95% CI**	**p-value**
Total Cough per loge(0.25-0.50 Hz) signal	1.13	1.06 to 1.20	< 0.001
**Day of Week**			
Sunday	(ref)	-	-
Monday	1.73	1.04 to 2.88	0.036
Tuesday	1.45	0.85 to 2.49	0.174
Wednesday	1.51	0.89 to 2.57	0.130
Thursday	1.43	0.84 to 2.44	0.188
Friday	1.33	0.78 to 2.29	0.296
Saturday	1.46	0.87 to 2.47	0.155

### 4.3 Comparing with body temperature based surveillance

Traditionally, “Fever (> 38*C*°)” is used as a biomarker for screening respiratory illnesses. However, in our data, the number of patients with fever “Fever (> 38*C*°)” fail to achieve any statistically significant correlation with daily positive COVID cases as shown in Table 2. The ‘Total Cough' count, on the other hand, achieves a higher statistically significant correlation with postive COVID count (ρ = 0.40, *p* < 0.001). To compare the effect of these two measurements as feature in our COVID models, we used two different feature sets for training random forest-based model since it performed better than most of the other models we tried. Both of these sets contain the features “Total Speech,” “std_energy.” The main difference is in the third feature which is “Fever(> 38^*o*^*C*)” and “Total cough” respectively for the two sets. We can see from Table 5 that models with the “Total cough” feature perform notably better than models with “Fever(> 38^*o*^*C*)” for the three target variables across all the evaluation metrics.

**Table 5:** Leave one day out cross-validation results with Random Forest model with two different feature sets; fs1 indicates {“Fever(>38C)”, “Total Speech”, “std_energy”} and fs2 indicates {“Total cough”, “Total Speech”, “std_energy”}.

**Target variable**	**MAE**		**RMSE**		**Pearson corr**	
	**fs1**	**fs2**	**fs1**	**fs2**	**fs1**	**fs2**
Positive COVID (#)	2.26	1.78	3.44	2.53	0.03	0.43
COVID/People ratio(%)	0.96	0.75	1.47	1.05	0.05	0.49
COVID/Test ratio(%)	4.5	3.65	6.13	5.2	0.15	0.38

### 4.4 Feature importance analysis

**Figure 4:**
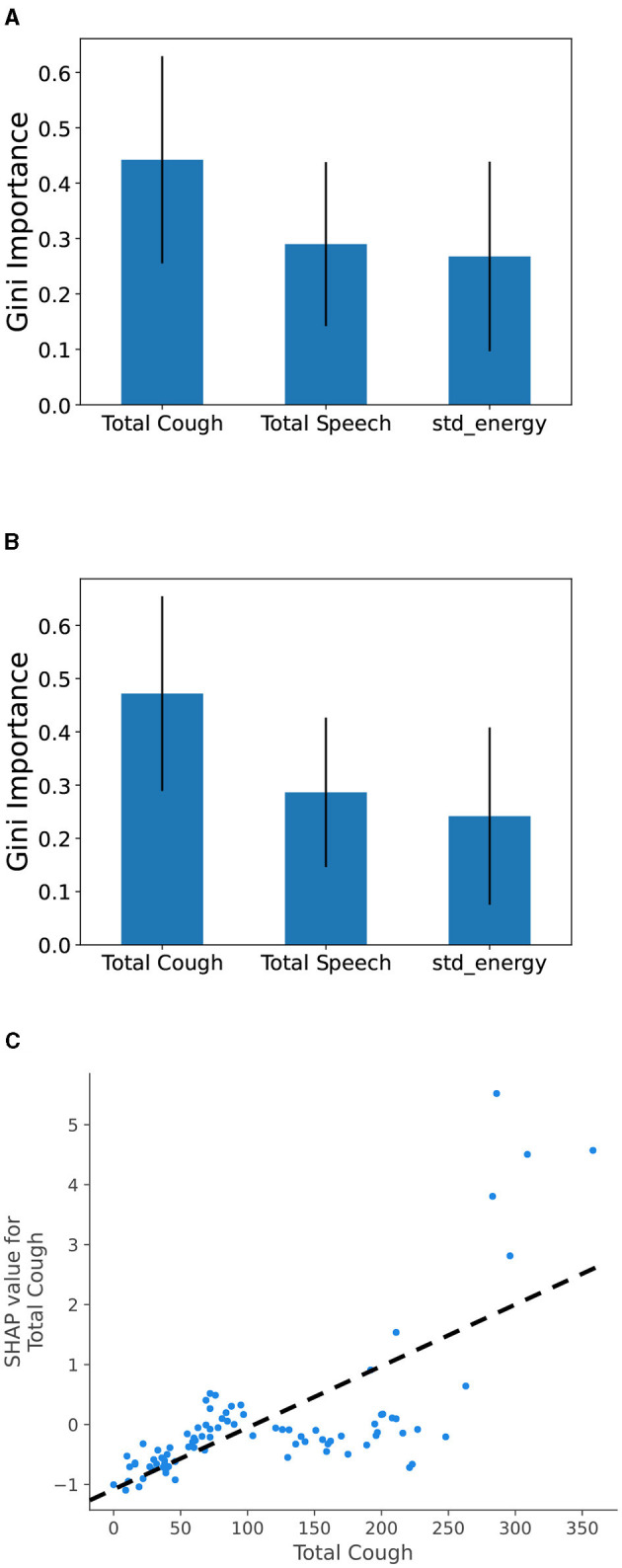
Gini feature importance for COVID burden estimation models: **(A)** modeling total Positive COVID cases and **(B)** modeling COVID-positive cases as fraction of the number of people, and **(C)** SHAP value for Total Cough feature. Overall, it shows that the increase in the total cough metric leads to a positive change (i.e., increase) in total COVID burden prediction.

We analyze the importance of the features in our best performing models to get a clear idea of which features play the most important role in the model prediction. Figure 4 shows Gini feature importance in our models for predicting COVID related statistics. We can observe that “Total Cough” has the highest importance for predicting both positive COVID count and positive COVID count as fraction of total number people visiting the hospital. This shows the prominence of total cough as feature in the models.

To compare with body temperature-based feature, we trained a Random Forest model using four specific features: “Total Cough,” “Total Speech,” “std_energy,” and “Fever (> 38^*o*^*C*),” to predict total COVID cases. To demonstrate the impact of features on model output, we utilized SHapley Additive exPlanations (SHAP) with TreeExplainer (41). SHAP analysis helps determine how individual feature values impact the output of a model. We observe similar pattern for the “Fever (> 38^*o*^*C*)” feature, however it contributes to a lesser extent compared to “Total Cough.” From Figure 4C, we can observe that high values for the cough count correspond to increased SHAP values. Overall, it indicates that the predicted COVID values tend to increase as the total cough increases.

## 5 Discussion

Our analysis revealed a significant and positive correlation between aggregated cough count and the total number of COVID-19 cases. Cough count exhibited a much stronger correlation compared to other syndromic indicators, such as the count of individuals presenting with fever. We also observed a strong correlation between the total number of individuals visiting the emergency department waiting room and the count of positive COVID cases. We demonstrated how cough count, along with other signals from our *Syndromic Logger* platform, could be useful for modeling the daily COVID case counts by developing regression models. Notably, the inclusion of cough count as a feature yielded the highest model performance boost as we observed with respect to multiple evaluation metrics. Feature importance analysis of our models highlighted cough count as the most influential predictor, based on Gini importance and SHAP value metrics.

Our results contribute to the growing body of evidence on how syndromic surveillance can improve our resilience to emerging public health crises (such as the COVID-19 pandemic and potential future outbreaks of similar infectious diseases). But they also provide compelling evidence that monitoring aggregated cough count serves as a reliable syndromic signal, which has the potential to complement existing population-level syndromic surveillance methods. The paper presents the design and architecture of a low-cost computational platform *Syndromic Logger* platform, which can be used for passive monitoring of total cough count in a public space (e.g., a public waiting room) in a privacy-sensitive manner. Currently, the majority of syndromic surveillance is conducted by collecting data directly from Electronic Health Record (EHR) systems. This data aggregation from multiple hospital networks is complex and limited in scale. Other alternatives, such as social media analysis, do not capture syndromic signals directly, rendering them unreliable (42). With our current platform, we can monitor and capture COVID-19 or other syndromic signals directly from the crowd with greater granularity.

While body temperature screening has been widely adopted in hospitals and public spaces as a preventive measure against the spread of COVID-19 and similar diseases, our analysis reveals a stronger correlation between COVID burden and aggregated cough count compared to the count of individuals with fever. This finding aligns with previous observations noted in the literature (43–45). This underscores the significance of monitoring cough count related signals, which can be accomplished in a contactless and unobtrusive manner using our *Syndromic Logger* platform. By highlighting the association between aggregated cough count and COVID-19 burden, our finding emphasizes the importance of incorporating this signal into public health surveillance strategies which is consistent with previous findings such as Al Hossain et al. (3).

The monitoring of cough count holds significant relevance as a syndromic signal, and several key advantages make it highly advantageous to track. The measurement of cough count can be done with very high accuracy using machine learning-based models, which we discuss in detail in the methods section. With this capability, monitoring cough count provides a more objective signal compared to other signals such as trends in search engines or social media websites. Unlike the aforementioned signals, monitoring of cough offers several distinct advantages. It can be achieved without requiring active human participation or active effort. Our proposed approach enables real-time insights without any reporting delays. Additionally, we prioritize the security of the captured data and the preservation of privacy by collecting only aggregated information.

However, our study is not without limitations. One such limitation is its use in a hospital's emergency room waiting area, selected due to the convenience of accessing ground-truth from the hospital Electronic Health Record (EHR) data. In this setting, every patient was screened for COVID-19, simplifying the acquisition of ground truth for our target population and enhancing our research findings' reliability. However, the characteristics and proportion of patients, including those suffering from COVID-19 and/or other respiratory diseases, differs from other outpatient settings, potentially introducing bias into our study. Additionally, our results were validated in only a single season and setting, constrained by the challenges of deploying and collecting data across multiple locations and timescales. We also anticipate variability in the predominant virus strains over time [e.g., the Delta variant (46)], which may exhibit different syndromic profiles compared to other SARS-CoV-2 variants (47, 48). Another potential limitation arises from using cough as a primary indicator as it is not necessarily specific to respiratory illnesses. Cough based syndromic surveillance will also fail to capture asymptomatic occurrence and transmission of COVID cases. Also, the prevalent cause of coughing may vary seasonally due to factors such as allergens, flu outbreaks, or other respiratory disease outbreaks, therefore, there will be variations in the frequency of coughing across different seasons, such as summer and winter. Furthermore, our present research does not differentiate between various cough types, such as dry or wet coughs, nor does it examine the correlation between individuals who cough frequently and those who do not. Consequently, a multi-year, multi-location study protocol with a multivariate approach with other factors and variables, including air quality index, community-level demographics, comorbidity information and various cough related classifications, may be necessary to fully develop and validate cough-based syndromic surveillance.

Our system comprises affordable components, facilitating its scalability for public health monitoring and multi-location longitudinal studies. The cough sensing part of our system (excluding the thermal camera and radar) costs less than 50 USD in today's market which lend itself well for potential large-scale deployment in a developing country scenario. Prior to large-scale deployment, it is essential to establish a proper cloud-based infrastructure for monitoring and aggregating data from these sensors. Additionally, maintaining edge devices pose a significant challenge, potentially requiring dedicated manpower to effectively address issues arising from such large-scale deployment. Geographical diversity may also present challenges in power and connectivity. However, with the increasing affordability and power efficiency of edge computing devices and deep-learning accelerators (49), future deployments of such platforms at scale are likely to be more cost-effective, even with low power requirements (e.g., using a battery pack or alternative power sources). Continuous improvement should also be feasible through insights gathered from real-world deployments, enhancing the reliability of our system. Additionally, to integrate our system efficiently with various governmental bodies, aspects such as data standardization and interoperability, secure data transmission, API management, and a continuous feedback collection system for governmental bodies should be considered. Establishing a pipeline for training and support is also essential to facilitate proper integration with these bodies. One such example of how this type of system can be integrated with governmental bodies can be found in Valentim et al. (50). This study demonstrates how a syndromic surveillance platform can be integrated into a broader information technology ecosystem during a pandemic. Given that our current *Syndromic Logger* can collect data in real-time while maintaining privacy at a more granular level, it has the potential to enhance any governmental response system in the future.

## 6 Conclusion

Based on our findings, we propose the integration of a sensor-based, contactless, and unobtrusive platform as a valuable augmentation to existing approaches, such as body temperature-based screening, in diverse public environments. Our results demonstrate that sensor-based platforms have the ability to capture clinically relevant syndromic signals, specifically the aggregated cough count. This capability allows us to overcome the limitations of existing methods which include reliance on manual human logging and reporting, as well as associated reporting delays. By embracing the adoption of sensor-based platforms, we can enhance the effectiveness and efficiency of public health surveillance, providing a superior solution for detecting and monitoring public health situations to overcome potential challenges in the future.

## Data availability statement

De-identified daily aggregated syndromic data will be made freely available, to qualified academic investigators for non-commercial research as required by the National Science Foundation and National Institutes of Health (NIH) Grants Policy on Sharing of Unique Research Resources and as permitted by the UMass IRB. Investigators must submit a formal request for data to the Principal Investigator who will grant permission to release the data as long as it meets the following requirements: (1) institution-specific permission to use the data for research, (2) guarantee that data will be used for research purposes only, and (3) completion of a standard data use agreement. Requests to access the datasets should be directed to trahman@ucsd.edu.

## Ethics statement

Ethical approval was not required for this study because no individual level data or individually identifiable information was collected. UMass IRB determined that the research project is not human subject research and does not meet the definition of human subject research under federal regulations [45 CFR 46.102(d)]. Written informed consent for participation was not required from the participants or the participants' legal guardians/next of kin in accordance with the national legislation and institutional requirements.

## Author contributions

FA: Conceptualization, Data curation, Formal analysis, Investigation, Methodology, Project administration, Software, Validation, Visualization, Writing – original draft, Writing – review & editing. MT: Data curation, Formal analysis, Investigation, Validation, Visualization, Writing – original draft, Writing – review & editing. SN: Conceptualization, Data curation, Resources, Writing – review & editing. BC: Conceptualization, Project administration, Writing – review & editing. RG: Supervision, Validation, Writing – review & editing. AL: Formal analysis, Investigation, Supervision, Validation, Writing – review & editing. RD: Supervision, Writing – review & editing. SC: Conceptualization, Funding acquisition, Project administration, Resources, Supervision, Writing – review & editing. TR: Conceptualization, Formal analysis, Funding acquisition, Investigation, Methodology, Resources, Supervision, Validation, Writing – original draft, Writing – review & editing.
